# Factors associated with an increased risk of vertebral fracture in monoclonal gammopathies of undetermined significance

**DOI:** 10.1038/bcj.2015.71

**Published:** 2015-08-28

**Authors:** J M Piot, M Royer, A Schmidt-Tanguy, E Hoppé, M Gardembas, T Bourrée, M Hunault, S François, F Boyer, N Ifrah, G Renier, A Chevailler, M Audran, D Chappard, H Libouban, G Mabilleau, E Legrand, B Bouvard

**Affiliations:** 1 Department of Rheumatology, Angers University Hospital, Angers, France; 2 Department of Hematology, INSERM U892/CNRS 6299, Angers University Hospital, Angers, France; 3 Laboratory of Immunology and Allergology, Angers University Hospital, Angers, France; 4 GEROM Research Group on Bone Remodeling and bioMaterials, UPRES EA 4658, University Hospital, Angers, France

## Abstract

Monoclonal gammopathies of undetermined significance (MGUS) have been shown to be associated with an increased risk of fractures. This study describes prospectively the bone status of MGUS patients and determines the factors associated with vertebral fracture. We included prospectively 201 patients with MGUS, incidentally discovered, and with no known history of osteoporosis: mean age 66.6±12.5 years, 48.3% women, 51.7% immunoglobulin G (IgG), 33.3% IgM and 10.4% IgA. Light chain was kappa in 64.2% patients. All patients had spinal radiographs and bone mineral density measurement in addition to gammopathy assessment. At least one prevalent non-traumatic vertebral fracture was discovered in 18.4% patients and equally distributed between men and women. Fractured patients were older, had a lower bone density and had also more frequently a lambda light chain isotype. Compared with patients with κ light chain, the odds ratio of being fractured for patients with λ light chain was 4.32 (95% confidence interval 1.80–11.16; *P*=0.002). These results suggest a high prevalence of non-traumatic vertebral fractures in MGUS associated with lambda light chain isotype and not only explained by low bone density.

## Introduction

Monoclonal gammopathy of undetermined significance (MGUS) is an asymptomatic plasma cells disorder occurring in 3% of adults >50 years old and 8% of adults >85 years old.^1^ MGUS is often fortuitously discovered and is detected by the electrophoresis of serum or urine protein, confirmed and typed by immunoelectrophoresis or immunofixation of serum and/or urine protein. In most cases, MGUS do not become malignant B-cell disorders.^2^ Although bone consequences of multiple myeloma (MM) are well known,^3^ the effects of MGUS on bone remodeling remain uncertain. Previous histological, laboratory and mainly clinical evidence have already shown that MGUS is a risk factor of fracture and particularly increases the risk of vertebral fractures. According to retrospective studies, MGUS is associated with an increased risk of fracture at any site of 1.4–2.5 times greater than in control populations and the risk of vertebral fracture up to 6 times greater.^4, 5^ Matthew Drake recently proposed to replace the term 'monoclonal gammopathy of undetermined significance' in favor of the term 'monoclonal gammopathy of skeletal significance' in order to more accurately reflect the enhanced skeletal risks inherent in this condition.^6^ However, factors associated with increased risk of fracture associated with MGUS remains poorly understood. Furthermore, the previous retrospective studies did not distinguish between traumatic and non-traumatic vertebral fractures, and the lack of systematic radiographic evaluation can lead to an underestimated number of vertebral fractures. Previous studies have demonstrated that nearly 50% of vertebral fractures are asymptomatic and that 30–50% of non-traumatic vertebral fractures occur in patients with intermediate bone mineral density (BMD) (–2.5<T-score>–1).^7, 8, 9^ So that only systematic spinal radiographs allow to detect all fractures and to evaluate the severity of each fracture. The detection of non-traumatic vertebral fractures is crucial because their number and their severity are major predictors of further vertebral and non-vertebral fractures.^10, 11^ Subjects with prevalent vertebral fracture have a fivefold increased risk of further vertebral fracture and a threefold increased risk of hip fracture.^12^ The last consensus about MGUS evaluation recommend assessing BMD at the initial evaluation but do not mention any systematic vertebral fracture research.^13, 14^ The aim of our study was to evaluate prospectively the bone and hematological status of patients with MGUS, to determine vertebral fracture prevalence in this population with systematic spinal radiographs and to identify risk factors for vertebral fracture.

## Patients and methods

### Patients

This prospective and descriptive study was conducted by both the Department of Rheumatology and the Department of Blood Diseases of the University Hospital of Angers, France, from 1 January 2008 to 30 April 2013. Patients were referred by general practitioners.

Patients included were adults and had a monoclonal gammopathy confirmed by immunoelectrophoresis of serum or urine protein. Monoclonal gammopathy had to be discovered in other situations than osteoporosis assessment. Mostly the electrophoresis was made in the medical check-up of asthenia or inflammatory syndrome or arthralgia. Patients with monoclonal gammopathy discovered during osteoporosis or fracture assessment, patients with previous known and treated osteoporosis and patients known to have a previous history of traumatic vertebral fractures were excluded. At the end of the bone and hematological assessment, patients with a diagnosis of hematologic malignancy were also excluded (symptomatic or asymptomatic MM or Waldenström macroglobulinemia, chronic lymphocytic leukemia). Finally, we included only patients with MGUS defined as International Myeloma Working Group criteria by (a) a serum monoclonal immunoglobulin concentration below 3 g/dl; (b) the absence of lytic bone lesions or pathological fractures; (c) the absence of anemia, hypercalcemia or renal insufficiency; and (d) a proportion of plasma cells in the bone marrow below 10%.^15^

The study was carried out in accordance with the ethical standards set by the Declaration of Helsinki. The entire study protocol was approved by the local ethical committee. Patients gave their informed consent.

### Methods

All the following data were collected for each patient. All the exams were performed the same day for one patient.

#### Clinical data

Interrogation and clinical data examination were performed to each patient to collect the following information: age, weight, height, body mass index (BMI), comorbidities, age at onset of menopause, hormonal replacement therapy, family history of fractures, personal history of fractures and their incidence condition, current treatment and calcium dietary intake.

#### Spinal radiographs and vertebral fracture assessment

Each patient had antero-posterior and lateral thoracic and lumbar spine radiographs. Two trained investigators (at least a 10-year experience in rheumatology and bone diseases) who were unaware of the patient BMD status, analyzed radiographs independently in three steps: (a) detect vertebral fracture; (b) determine benign or malignant origin; and (c) classify the fracture as mild, moderate or severe.

The patient was classified as having a vertebral fracture if both readers independently found a definite fracture. He/she was classified as normal if both readers independently found that the films were normal. When the readers disagreed, the films were reviewed in conference by both investigators. Each vertebral fracture was classified as benign or malignant using classical radiographic signs (destruction of the cortical margin or not, posterior convexity or not). The benign vertebral fractures were characterized by the semiquantitative classification of Genant and defined as such: *mild* or *grade 1* for a reduction of 20–25% of anterior, middle and/or posterior height; *moderate* or *grade 2* for a reduction of 26–40% in any height; and *severe* or *grade 3* for a reduction >40% in any height.^16^

#### Bone mineral density

BMD was measured using dual energy X-ray absorptiometry operating in fan-beam mode (Hologic QDR 4500A densitometer, Hologic Inc., Waltham, MA, USA). Quality control scans were carried out daily, using the manufacturer-supplied anthropomorphometric spine phantom; the long-term (>1 year) coefficient of variation was 0.40%. Lumbar BMD was assessed from L2 to L4, in the postero-anterior view incidence, and fractured vertebrae were excluded from the analysis. Total hip BMD and femoral neck BMD were measured at upper left femur. The mean precision error of dual energy X-ray absorptiometry measurement is <1.5% for the lumbar spine and <2% for hip BMD. As usually, the results were expressed in absolute values (g/cm^2^) and using the T-score. The T-scores were calculated using manufacturer's references and expressed the difference between the subject value and the mean value of healthy young adults. The World Health Organization has defined normal BMD as a T-score >−1, low bone density as a T-score between −2.5 and −1, and osteoporosis as a T-score <−2.5.

#### Biological data

Laboratory tests were performed on fasting subjects at 0800 hours without freezing to confirm and quantify gammopathy and to assess parameters of mineral metabolism and bone turnover: serum protein electrophoresis to obtain the monoclonal protein level, serum and urinary immunoelectrophoresis, cells blood count, β2 microglobulin, lactate dehydrogenase, creatinine, bone marrow by sternal puncture in patients with immunoglobulin G (IgG) or IgA isotype, bone marrow biopsy in patients with IgM isotype having a visible protein electrophoresis peak, serum calcium, phosphate, albumin, 25-hydroxyvitamin D, parathyroid hormone, bone-alkaline phosphatase and C-terminal telopeptide of type I collagen serum.

### Statistical analyses

Statistical analysis was performed using the software Statistical Package for the Social Sciences (SPSS V.15.0.1, SPSS Inc., IBM Corporation, Chicago, IL, USA). Baseline characteristics of patients were expressed in mean±1 s.d. for continuous variables and *n* (%) for categorical variables. The comparison of groups was performed for continuous variables by analysis of variance and for binary variables by the Pearson *X*^2^. Differences were considered significant when *P*<0.05. Logistic regression was performed to analyze factors associated with at least one vertebral fracture. As number of vertebral fractures is a criterion of fracture severity, logistic regression was also performed to analyze factors associated with at least two vertebral fractures. Multivariate analyses comprised age, sex, BMI, low BMD with a T-score <–2.5, heavy chain isotype and light chain isotype.

## Results

### Characteristics of the population

**Figure 1:**
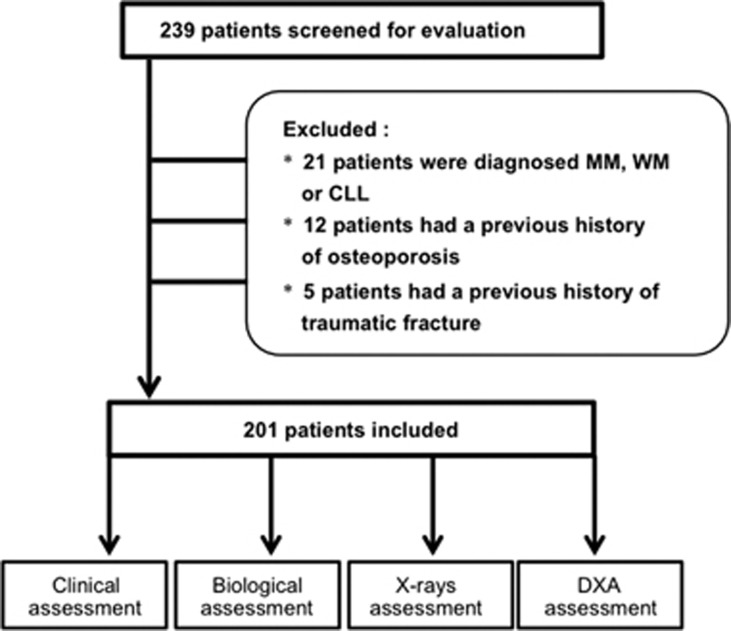
Flowchart. Abbreviations: MM, multiple myeloma; WM, waldenström's macroglobulinemia; CLL, chronic lymphocytic leukemia; DXA, dual energy X-ray absorptiometry.

Flowchart of the study is detailed in Figure 1 and characteristics of the population in Table 1. Two hundred one patients were included, 97 women (48.3%) and 104 men (51.3%). The average age was 66.6±12.5 years (range: 30-89 years), and the mean BMI was 26.7±5.1 kg/m^2^. One hundred ninety-five patients (97%) were of Caucasian ethnicity. The distribution of heavy chain isotypes was: 104 IgG (51.7%), 67 IgM (33.3%), 21 IgA (10.4%), nine dual isotype (4.5%) and no light chain MGUS. The light chains were distributed as follows: 129 κ light chains (64.2%), 63 λ light chains (31.3%) and nine κ+λ associations (4.5%). The average monoclonal protein was 5.98±4.87 g/l (range: 0–21.3 g/l) and the mean plasma cells in bone marrow of 3.3±2.3% (range 0–9%). Thirty-six patients (17.9%) had a BMD T-score <−2.5 in at least one of the three measured sites. Thirty-seven patients (18.4%) had at least one prevalent vertebral fracture at the thoracic or lumbar spine. None of these fractures was tumoral or traumatic. Seventy-six vertebral fractures were detected, 21 grade 1, 40 grade 2 and 15 grade 3. Twenty patients had one vertebral fracture; six patients had two vertebral fractures, six patients had three vertebral fractures and five patients had four or more vertebral fractures. The prevalence of vertebral fracture was 10% for patients under 65 years old, 13% for patients between 65 and 74 years old and 33% for patients aged of 75 years old and more. Sixty-two percent of patients with a vertebral fracture had no densitometric osteoporosis with a T-score ⩾−2.5 at the spine and the hip.

### Factors associated with vertebral fracture

The 37 patients with vertebral fracture(s) were significantly older than non-fractured (73.54 vs 65.07 years; *P*<0.001) and had a significantly lower T-score and BMD regardless of the studied site. There was no significant difference in sex ratio, BMI or distribution of heavy chain isotypes between groups. Patients with vertebral fracture had significantly more λ light chain isotype than patients without fracture (48.65% vs 27.44% *P*=0.01) (Table 2). In univariate and multivariate analyses, age, low BMD and λ light chain isotype were associated with a significantly increased risk of vertebral fracture (Table 3). Compared with patients with κ light chain, the odds ratio of being fractured for patients with λ light chain was 4.32 (95% confidence interval 1.80–11.16; *P*=0.002). We did not observe any biological difference between fractured and non-fractured patients in terms of 25 hydroxyvitamin D, PTH, calcemia, β2 microglobulin, monoclonal protein value and marrow cells count.

### Factors associated with ⩾2 vertebral fractures

As number of vertebral fractures is a criterion of fracture severity, we analyzed factors associated with ⩾2 vertebral fractures (Table 4). Seventeen patients (8.4%) were concerned. In univariate analysis, age, BMD <−2.5 and λ isotype light chain were associated with a significant risk of ⩾2 vertebral fractures. In multivariate analysis, low BMD and λ isotype light chain remained significantly associated with the risk of ⩾2 vertebral fractures. A higher BMI was also significantly associated with the presence of ⩾2 vertebral fractures. There was no difference between patients with λ isotype light chain and patients with κ isotype light chain in terms of sex, age, BMI, BMD or biological parameters.

## Discussion

In this study, we conducted a prospective bone evaluation in 201 men and women with MGUS. The population of this study is representative of a MGUS population, with a sex ratio close to 1 (48.3% women), an isotype distribution of heavy and light chains (IgG 51.7%, 33.3% IgM, 10.4% IgA, biclonal 4.5%, κ chain 64.2% and λ chain 31.3%) similar to what is usually described in MGUS cohort studies of western France albeit with a greater percentage of IgM compared with north American registry studies.^5, 17, 18^ To better define the risk factors associated with vertebral fracture in MGUS, we selected patients with strict criteria, excluding patients with hematological diseases, known osteoporosis or a history of vertebral fracture. All patients had spine radiography, which are the most reliable means to detect vertebral fracture, especially asymptomatic ones. Two trained investigators have read spine radiography in this study to be sure to detect all vertebral fractures, to diagnose pathological fractures and to eliminate simple vertebral deformity, which sometimes can be confused with mild vertebral fractures.

The first observation was the high number of prevalent vertebral fractures in our cohort (18.4%). Seventy-two percent of fractures were grade 2 or 3, and multiple vertebral fractures were detected in 45% of the fractured patients. We do not have a control group to compare the prevalence of vertebral fracture of MGUS patients with the general population. Data from the European Vertebral Osteoporosis Study (EVOS), a multicenter multipopulation survey of vertebral osteoporosis included 15 570 males and women aged 50–79 years, have shown that the standardized population prevalence of morphometrically vertebral deformity across Europe was 12.2% for men and 12.0% for women.^19^ This result indicates that the discovery of vertebral fractures is frequent in monoclonal gammopathy even in absence of MM or Waldenström macroglobulinemia. Patients with vertebral fracture were significantly older and had a lower spine and hip BMD, two known risk factors for osteoporotic fractures. However, some characteristics associated with vertebral fractures were unusual. In our study, prevalent fractures were as frequent in men as in women, a part of vertebral fractures occurred also before 65 years, and patients with high BMI were exposed to multiple fractures. These features strongly contrast with the characteristics of osteoporosis, that is, a more frequent prevalence of vertebral fractures in women, after 65 years and in patients with low BMI. These results strongly suggest another mechanism than osteoporosis to explain vertebral fracture in this population. An important role of bone quality with an altered bone microstructure in MGUS has been recently suggested.^20^

The second main result is the significant association between the presence of the λ light chain isotype and the presence of vertebral fractures with an odds ratio of vertebral fracture of 4.32 in the λ group compared with the κ group. The association between vertebral fracture and λ light chain remains significant in multivariate analysis after adjustment on BMD, age, sex and BMI. The association between λ light chain and vertebral fractures remains also significant when we analyzed patients with at least two vertebral fractures. Previous studies have shown contradictory results concerning the influence of isotypes on fracture risk.^4, 5, 21, 22, 23^ Melton *et al.*^5^ have found a protective effect of IgG isotype whereas Kristinsson *et al.*^22^ found no influence of heavy or light chain isotype on the risk of fracture. With regard to our study, we do not have any explanations concerning the link between the λ light chains and the risk of vertebral fracture. The κ and λ light chains have different chemical properties and despite the number of potential combinations of light chain because of the genetic rearrangement, some subtypes are more frequently associated with deposit diseases.^24^ The immunoglobulin light chain amyloidosis and POEMS syndrome are most commonly linked with λ light chains, whereas myeloma with renal involvement is more common in the presence of κ light chains.^25, 26, 27^ Our study describes another potential λ light chain-associated disease.

Our study shows a high prevalence of vertebral fracture in MGUS, which is not completely explained by osteoporosis. MGUS represents a potentially pre-neoplastic condition that may progress to malignant B-cell disorders, such as MM. In MM, bone lesions are due to the secretion of many cytokines by plasma cells, bone marrow stromal cells, osteoblasts and osteoclasts, leading to an uncoupling of bone remodeling with increased bone resorption contrasting with a reduction in bone formation.^28, 29, 30^ Some studies have shown a similar cytokine profile in MGUS with an increased cytokine serum levels such as Dickkopf-related protein 1 and Macrophage inflammatory protein 1α and an increased Receptor activator of nuclear factor kappa B ligand/Osteoprotegerin ratio in patients MGUS with and without osteoporotic vertebral fractures.^31, 32^ Bone turnover in monoclonal IgM gammopathy seems to be related to a different mechanism with an increased microresorption because of a population of mononucleated osteoclasts.^33^

In summary, in this prospective study of 201 men and women with well-defined MGUS, we observed a high prevalence of non-traumatic vertebral fractures, equally distributed between men and women, and strongly associated with the presence of a λ light chain. The mechanism of these fractures is currently unknown and may be not only limited to low bone density. The significance of these fractures on the future of MGUS needs further prospective studies. These results confirm the previously published data and reinforce the concept of the need for bone assessment of all patients with MGUS with systematic vertebral fracture assessment in addition to BMD.

## Figures and Tables

**Table 1:** Characteristics of the population (201 patients)

*Characteristics*	*Mean value*±*s.d.*
Age, years	66.63±12.49
Weight, kg	72.35±14.54
Height, cm	164.06±9.13
BMI, kg/m^2^	26.73±5.1
BMD total hip, g/cm^2^	0.897±0.154
T-score total hip, s.d.	−0.72±1.01
BMD femoral neck, g/cm^2^	0.738±0.14
T-score femoral neck, s.d.	−1.30±1.05
BMD lumbar spine, g/cm^2^	0.958±0.163
T-score lumbar spine, s.d.	−1.09±1.45
Albumin, g/l (nl: 40–47)	42.36±5.01
Calcium, mmol/l (nl: 2.10–2.43)	2.33±0.11
Creatinine, μmol/l (nl: 64–104)	77.31±24.79
25-Hydroxyvitamin D, nmol/l (nl: 75–250)	55.47±28.48
PTH, pg/ml (nl: 15–65)	38.06±24.08
β2 Microglobulin, mg/l (nl: 1.20–2.50)	2.22±0.98
LDH, UI/l (nl: 125–220)	278.51±108.75
CTX, ng/ml	0.64±1.31
BALP, UI/l (nl: 5.5–24.6)	13.75±8.89
Marrow plasma cells (%)	3.3±2.33
Peak value, g/l	5.9±4.8
IgA	4.2±3.6
IgG	6.3±5.3
IgM	5.9±4.3
Double peak	7.2±6.4
	
*Categorical variables*	
Women	97 (48.3%)
α Heavy chain	21 (10.4%)
γ Heavy chain	104 (51.7%)
μ Heavy chain	67 (33.3%)
Double heavy chain isotype	9 (4.5%)
κ Light chain	129 (64.2%)
λ Light chain	63 (31.3%)
Double light chain isotype	9 (4.5%)

Abbreviations: BALP, bone-alkaline phosphatase; BMD, bone mineral density; BMI, body mass index; CTX, C-terminal telopeptide of collagen-1; Ig, immunoglobulin; LDH, lactate dehydrogenase; nl, normal; PTH, parathormone.

**Table 2:** Characteristics of the population according to fractured status

*Characteristics*	*Fractured (*N= *37)*	*Not fractured (*N= *164)*	P-*value*
	*Mean*±*s.d.*	*Mean*±*s.d.*	
Age (years)	73.54±10.28	65.07±12.47	<0.001
BMI (kg/m^2^)	27.27±4.73	26.75±4.75	0.558
BMD total hip (g/cm^2^)	0.789±0.114	0.920±0.152	<0.001
T-score total hip (DS)	–1.40±0.76	–0.56±0.99	<0.001
BMD femoral neck (g/cm^2^)	0.636±0.094	0.759±0.139	<0.001
T-score femoral neck (DS)	–1.96±0.75	–1.14±1.04	<0.001
BMD lower spine (g/cm^2^)	0.851±0.121	0.974±0.161	0.005
T-score lower spine (DS)	–2.09±1.10	–0.92±1.43	0.001
Albumin (g/l)	40.74±5.07	42.75±4.94	0.027
Calcium (mmol/l)	2.33±0.12	2.33±0.10	0.964
Phosphate (mmol /l)	1.03±0.13	0.99±0.16	0.217
Creatinine (μmol/l)	80.19±27.49	76.66±24.26	0.437
C-reactive protein (mg/l)	12.11±16.78	7.43±16.23	0.118
25 Hydroxyvitamine D (nmol/l)	61.89±34.67	54.22±26.93	0.150
PTH (pg/ml)	36.89±27.06	38.36±23.52	0.745
β2 Microglobulin (mg/l)	2.49±0.89	2.16±0.99	0.075
LDH (UI/l)	287.54±104.57	276.56±110.21	0.591
Marrow plasma cells (%)	3.13±2.16	3.34±2.37	0.752
Monoclonal protein in serum (g/l)	6.95±5.32	5.75±4.78	0.180
BALP (UI/l)	14.94±7.03	13.49±9.29	0.379
			
*Categorical variables*			
Sex (women)	20 (54.05%)	83 (50.61%)	0.73
γ Heavy chain (IgG)	16 (43.24%)	88 (53.66%)	0.27
μ Heavy chain (IgM)	17 (45.94%)	50 (30.49%)	
α Heavy chain (IgA)	2 (5.41%)	19 (11.59%)	
Double isotype heavy chain	2 (5.41%)	7 (4.27%)	
κ Light chain	16 (43.24%)	113 (68.90%)	0.013
λ Light chain	18 (48.65%)	45 (27.44%)	
Double isotype light chain	3 (8.11%)	6 (3.66%)	

Abbreviations: BALP, bone-alkaline phosphatase; BMD, bone mineral density; BMI, body mass index; CTX, C-terminal telopeptid of collagen-1; Ig, immunoglobulin; LDH, lactate dehydrogenase; PTH, parathormone.

**Table 3:** Factors associated with vertebral fracture in 201 patients

	*Odds ratio*	*95% Confidence interval*	P-*value*
*Univariate analysis*			
Age	1.07	1.03–1.11	0.001
Sex	1.13	0.55–2.32	0.730
BMI	1.02	0.95–1.10	0.560
Low BMD (T-score <−2.5)	4.19	1.85–9.50	0.001
IgM vs IgG and IgA	1.92	0.93–3.97	0.078
IgG vs IgM and IgA	0.67	0.32–1.37	0.270
IgA vs IgG and IgM	0.43	0.10–1.95	0.270
Lambda vs kappa	2.51	1.21–5.24	0.014
			
*Multivariate analysis*			
Age	1.07	1.03–1.12	0.002
Sex	1.08	0.45–2.58	0.860
BMI	1.03	0.94–1.13	0.470
Low BMD	3.65	1.44–9.26	0.006
Heavy chain isotype	1.67	0.87–3.19	0.120
Lambda vs kappa	4.32	1.80–11.16	0.002

Abbreviations: BMD, bone mineral density; BMI, body mass index; Ig, immunoglobulin.

**Table 4:** Factors associated with ⩾2 vertebral fractures

	*Odds ratio*	*95% Confidence interval*	P-*value*
*Univariate analysis*			
Age	1.05	1–1.10	0.033
Sex	1.06	0.39–2.88	0.900
BMI	1.11	1.01–1.23	0.026
Low BMD (T-score <−2.5)	3.57	1.25–10.19	0.017
IgM vs IgG and IgA	1.87	0.68–5.09	0.220
IgG vs IgM and IgA	0.48	0.17–1.36	0.170
IgA vs IgG and IgM	1.15	0.24–5.42	0.860
Lambda vs kappa	4.65	1.64–13.23	0.004
			
*Multivariate analysis*			
Age	1.05	0.29–3.13	0.086
Sex	0.86	0.26–2.80	0.800
BMI	1.15	1.03–1.29	0.014
Low BMD	4.11	1.20–14.08	0.025
Heavy chain isotype	1.59	0.63–4.00	0.320
Lambda vs kappa	6.09	1.77–20.92	0.004

Abbreviations: BMD, bone mineral density; BMI, body mass index; Ig, immunoglobulin.
